# Supplementary material for: Newly Diagnosed Crohn’s Disease Patients in India and Israel Display Distinct Presentations and Serological Markers: Insights from Prospective Cohorts
Source: J Clin Med. 2022 Nov 22;11(23):6899. doi: 10.3390/jcm11236899 (PMC9737641; doi:10.3390/jcm11236899)
Supplement: Supplementary file 1 [file jcm-11-06899-s001.zip › jcm-2036625-supplementary.pdf]

Journal of
Clinical Medicine

MDPI

Supplementary Materials

Newly Diagnosed Crohn's Disease Patients in India and Israel Display Distinct Presentations and Serological Markers: Insights from Prospective Cohorts

Supplementary Table S1. Susceptibility genes variation.

Gene Name	SNP ID	Assay ID
ATG16L1	rs2241880	C___9095577_20
ATG16L1	rs4663402	C__28001596_10
ATG16L1	rs4663421	C__27884541_10
NOD2	rs2066844	C__11717468_20
NOD2	rs2066845	C__11717466_20
NOD2	rs2066847	C__60383785_10
CARD8	rs2043211	C__11708080_1_
CARD9	rs10781499	C__25957125_10
NOX3	rs6557421	C___2468042_30
DUOX2	rs151261408	C_163885945_10
IRGM	rs11741861	C_176034574_10
IRGM	rs4958847	C___1398968_10
IRGM	rs1000113	C__27107152_10
IRGM	rs180802994	C_176206673_10
MHC	rs9279411	See probes below *
XACT	rs5929166	C__29023189_10
IGFBP	rs75764599	C_100812686_10
FOXO3	rs147856773	See probes below **
TNF-α	rs1799964	C___7514871_10
TNF-α	rs1799724	C__11918223_10
JAK2	rs1887428	C__12095830_20

* MHC_rs92794111# PrimerFW GGGAGCGGAGAGGAGGATTCTGA. PrimerRV TCTT-GCCCGGCCAATGCTTATCC. Probe FAM-CCTGCCCGTCCCCC. Probe VIC-CTGCCCCTGTCCCCC. ** FOXO3_rs1478567731# PrimerFW GTGG-CATTTTAATGTTCTCAGAACCA. PrimerRV GCCTGAACCTCAATTCCTGATTTC. Probe VIC-CAGTGTGATGTAAGAAA. Probe FAM-TGTGATGTGGTAAGAAA.

Supplementary Table S2. Comparison of demographic characteristics between patients with newly diagnosed complicated and uncomplicated CD phenotype in Indian and Israeli cohorts.

	Indian cohort (n=104)			Israeli cohort (n=156)		
	**Complicated phenotype[&] (n=50)**	**Uncomplicated phenotype (n=53)**	**P value**	**Complicated phenotype[&] (n=47)**	**Uncomplicated phenotype (n=109)**	**P value**
**Gender** (male), n(%)	16 (32)	20 (37)	0.681	23 (48.9)	54 (49.5)	0.999
**Age**, years ±SD	38.12±12.9	37.62±13.05	0.805	31.64±11.59	31.88±13.48	0.569
**BMI**, kg/m2±SD	20.5±4.19	19.42±4.39	0.205	22.76±4.18	23.74±4.6	0.213
**Active smokers**, n(%)	2 (4)	7 (13)	0.116	14 (29.8)	20 (19)	0.289
**Family history of Crohn's disease**, n(%)	1 (2)	3 (5.6)	0.619	9 (19.1)	26 (23.9)	0.541
**Extra-intestinal manifestations**, n(%)	15 (27.8)	7 (14)	0.098	13 (30.2)	40 (44)	0.136

[&] stricturing, and/or penetrating, and/or perianal Crohn's disease phenotype.
